# Editorial: Autonomic involvement in arrhythmias: translating mechanisms to therapy

**DOI:** 10.3389/fcvm.2024.1504473

**Published:** 2024-10-25

**Authors:** Stefanos Zafeiropoulos, Ioannis Doundoulakis, Stavros Zanos, Stavros Stavrakis

**Affiliations:** ^1^ Department of Cardiology, University Hospital of Zurich, Zürich, Switzerland; ^2^ Center for Translational and Experimental Cardiology, Zürich, Switzerland; ^3^ Postgraduate Program in Cardiac Electrophysiology and Pacing, Heart Rhythm Management Centre, University Hospital Brussels–Free University Brussels, European Reference Networks Guard–Heart, Brussels, Belgium; ^4^ Institute for Bioelectronic Medicine, Feinstein Institutes for Medical Research at Northwell Health, Manhasset, NY, United States; ^5^ Heart Rhythm Institute, University of Oklahoma Health Sciences Center, Oklahoma City, OK, United States

**Keywords:** autonomic nervous system, atrial fibrillation, ventricular arrhythmias, neuromodulation, ablation electrophysiology

**Editorial on the Research Topic**
Autonomic involvement in arrhythmias: translating mechanisms to therapy

The role of the autonomic nervous system (ANS) in arrhythmias has garnered considerable attention, with recent research underscoring its involvement in the initiation, maintenance, and progression of various cardiac arrhythmias (1). This special issue examines the intricate relationship between the ANS and arrhythmias, focusing on innovative diagnostic, therapeutic, and predictive strategies that are reshaping our understanding and management of these conditions. The contributions span basic research to clinical outcomes, offering comprehensive insights into this evolving field.

Vandenberk et al. have provided a comprehensive review of the role of the ANS in atrial fibrillation (AF), delivering deep insights into AF pathophysiology, which encompasses triggers, substrates, and remodeling processes. The review elucidates how the interplay between the sympathetic and parasympathetic nervous systems influences AF pathogenesis. Given the expanding array of therapeutic options, particularly the advent of pulsed-field ablation (PFA) technology—where inadvertent ganglionated plexi ablation may not occur to the same extent as with thermal ablation (2, 3)—their review provides valuable guidance on the non-invasive assessment of autonomic function. This includes using modern wearable devices, combined with artificial intelligence-based heart rate variability analysis, which could serve as a valuable tool to distinguish the vagal and adrenergic AF subtypes, thereby facilitating personalized AF management.

In the pursuit of more individualized AF treatment strategies, Ma et al. developed a novel algorithm to predict AF recurrence over a 2-year follow-up period in patients undergoing first-time radiofrequency ablation for atrial fibrillation. The model incorporated factors such as persistent AF, AF duration, left atrial diameter, estimated glomerular filtration rate, NT-proBNP, and autoantibodies against the M2-muscarinic receptor. This algorithm demonstrated superior predictive accuracy compared to existing scores, although external validation remains necessary.

Delving deeper into the field of AF ablation, Zuk et al. (4) investigated the association between ablation-induced baroreceptor reflex modification and procedural efficacy in patients with AF, providing novel insights into the neuromodulatory effects of thermal ablation methods, specifically cryoballoon and radiofrequency ablation. Importantly, the study demonstrated that both modalities led to a reduction in baroreceptor function, with a more pronounced decrease observed in the cryoballoon group. Despite these findings, no significant differences were observed in baroreflex parameters between patients with and without AF recurrence post-ablation, though the study was underpowered for this comparison. This work enriches our understanding of autonomic changes following ablation, but further research is warranted to thoroughly characterize the autonomic functional and structural alterations post-ablation and to assess their potential prognostic value. Investigating the emerging PFA into this context, which likely preserves most of the ganglionated plexi, could provide important mechanistic and clinical insights (2, 3).

Beyond AF, the role of the ANS is also critical in ventricular arrhythmias, with accumulating evidence suggesting that stellate ganglion blockade can inhibit ventricular fibrillation. Yu et al. delved into the molecular underpinnings of this phenomenon, examining the role of the Bmal1 gene—which modulates neural activity in the central nervous system—in ventricular arrhythmogenesis. Their study demonstrated that Bmal1 knockdown in the left stellate ganglion of beagles effectively suppressed neural activity and reduced ventricular arrhythmias following myocardial ischemia. These findings provide valuable insights into the molecular mechanisms involved and highlight potential therapeutic pathways for the management of ventricular arrhythmias.

Cardioneuroablation (CNA) has been proposed as a therapeutic intervention for patients with cardioinhibitory vasovagal syncope (5, 6). Extending this concept, Valenti et al. presented a compelling case report of a young patient with third-degree atrioventricular (AV) block who underwent CNA targeting the inferior paraseptal ganglionated plexus. The procedure resulted in complete resolution of the third-degree AV block and the patient's symptoms, suggesting that CNA might offer a personalized and effective treatment for AV node dysfunction in young patients, potentially serving as an alternative to pacemaker implantation. However, a randomized trials are needed in this patient's population.

Overall, the research presented in this special issue marks a significant advancement in our understanding of the autonomic nervous system's influence across the spectrum of arrhythmias, ranging from atrial fibrillation to ventricular arrhythmias and AV blocks. By exploring novel mechanistic pathways, diagnostic tools, predictive models, and therapeutic strategies, this issue highlights the potential to enhance patient outcomes and quality of life through targeted management approaches that address the autonomic component of arrhythmias. Continued investigation into the role of the ANS in arrhythmogenesis will undoubtedly be pivotal in the development of personalized treatment options for patients with arrhythmias.
